# Lysine Decarboxylase with an Enhanced Affinity for Pyridoxal 5-Phosphate by Disulfide Bond-Mediated Spatial Reconstitution

**DOI:** 10.1371/journal.pone.0170163

**Published:** 2017-01-17

**Authors:** Hye-Young Sagong, Kyung-Jin Kim

**Affiliations:** School of Life Sciences, KNU Creative BioResearch Group, Kyungpook National University, Daegu, Republic of Korea; Russian Academy of Medical Sciences, RUSSIAN FEDERATION

## Abstract

Lysine decarboxylase (LDC) catalyzes the decarboxylation of l-lysine to produce cadaverine, an important industrial platform chemical for bio-based polyamides. However, due to high flexibility at the pyridoxal 5-phosphate (PLP) binding site, use of the enzyme for cadaverine production requires continuous supplement of large amounts of PLP. In order to develop an LDC enzyme from *Selenomonas ruminantium* (*Sr*LDC) with an enhanced affinity for PLP, we introduced an internal disulfide bond between Ala225 and Thr302 residues with a desire to retain the PLP binding site in a closed conformation. The *Sr*LDC^A225C/T302C^ mutant showed a yellow color and the characteristic UV/Vis absorption peaks for enzymes with bound PLP, and exhibited three-fold enhanced PLP affinity compared with the wild-type *Sr*LDC. The mutant also exhibited a dramatically enhanced LDC activity and cadaverine conversion particularly under no or low PLP concentrations. Moreover, introduction of the disulfide bond rendered *Sr*LDC more resistant to high pH and temperature. The formation of the introduced disulfide bond and the maintenance of the PLP binding site in the closed conformation were confirmed by determination of the crystal structure of the mutant. This study shows that disulfide bond-mediated spatial reconstitution can be a platform technology for development of enzymes with enhanced PLP affinity.

## Introduction

l-lysine is an essential amino acid and industrially important material used in animal feed and food and dietary supplements. It can be synthesized from aspartate [1,2]. The aspartate is converted into l-aspartate semialdehyde (ASA) by the consecutive reaction of two enzymes. ASA is a precursor for the biosynthesis of various amino acids such as l-threonine, l-isoleucine, l-methionine, and l-lysine. On the l-lysine biosynthetic pathway, ASA is condensed with pyruvate to generate dihydrodipicolinate (DHDP). DHDP reductase reduces DHDP to produce tetrahydrodipicolinate (THDP). Currently, four different pathways for the biosynthesis of l-lysine that branch out from THDP have been reported in bacteria [3]: the succinylase pathway, the acetylase pathway, the *m*DAP dehydrogenase pathway, and the recently discovered aminotransferase pathway [4]. Finally, DAP decarboxylase catalyzes the decarboxylation of d,l-DAP to form l-lysine.

Lysine decarboxylase (LDC) is an important enzyme for maintenance of pH homeostasis and the biosynthesis of cadaverine. Most of bacteria utilize acid stress-induced lysine decarboxylase in the response to the environmental acid stress [5–7]. Under extremely low pH, LDC is induced and increases the pH of the growth medium by consuming one proton in the enzymatic reaction. In addition, LDC plays a crucial role in the synthesis of cadaverine, an important industrial platform chemical. Cadaverine is utilized with a variety of applications such as the production of polyamides, polyurethanes, chelating agents, and additives [8]. As the heavy consumption of petroleum resources contributes to the accelerating the global warming, it is necessary to find the alternative to previous petrochemical production of cadaverine. Bio-based production of cadaverine from renewable resources is a promising alternative in environmental and economic aspects [9,10]. Bio-based cadaverine is synthesized through the decarboxylation from l-lysine by LDC and enzymatic production using excessive addition of LDC in lysine-rich medium has been reported [11,12]. Especially, the most promising approach for bio-based supply of cadaverine is the use of genetically engineered microorganisms including *Escherichia coli* and *Corynebacterium glutamicum* with improved cadaverine yield [13–17].

However, two unsolved problems remain in the cadaverine production process. First, as LDC consumes one proton in the reaction, the pH of the medium increases [18]. In high pH, the activity of LDC dramatically decreases, which leads to the reduced cadaverine yield. One solution to this problem is development of the enzyme that retains enzymatic activity at high pH. Second, in the cadaverine production process, continuous supplement of pyridoxal-5-phosphate (PLP) is required as a cofactor due to the lack of PLP for LDC activity [15]. When PLP was added to the medium, LDC exhibits high level of activity and cadaverine yield. Many attempts are underway to reduce the additional cost of continuous supplement of PLP. Recently, a *de novo* PLP biosynthetic pathway was introduced into the cadaverine-producing strains to enhance the cellular level of PLP [19]. Recently, we determined the crystal structures of LDC from *Selenomonas ruminanitum* (*Sr*LDC) in different crystallization conditions and observed that *Sr*LDC contains highly flexible active site [20]. In addition, *Sr*LDC shows much lower PLP affinity than other PLP-dependent enzymes. Taken together, we propose that highly flexible active site contributes to the low affinity for PLP in *Sr*LDC.

In this study, to develop a highly efficient *Sr*LDC enzyme with an enhanced affinity for PLP, we applied disulfide bond-mediated spatial reconstitution by introducing a disulfide bond at the PLP binding site in *Sr*LDC. The mutant with an artificial disulfide bond showed enhanced PLP affinity and exhibited dramatically increased LDC activity and cadaverine conversion particularly under no or low PLP concentrations. The mutant also showed high resistance to both pH and temperature. This approach may be used as a platform technology for the development of other enzymes with enhanced PLP affinity.

## Materials and Methods

### Protein preparation

The gene coding for lysine decarboxylase from *S*. *ruminantium* (*Sr*LDC, amino acid residues 1–399) was amplified from chromosomal DNA of *S*. *ruminantium* by polymerase chain reaction (PCR) with primers: forward, 5-GCGCG**CATATG**AAAAACTTCCGTTTAAGCGAAAAAG -3, and reverse, 5-GCGCG**CTCGAG**GTGATGGTGATGGTGGTGAACTGCT -3. The PCR product was then subcloned into the pET-22b(+) vector (Life Science Research) with 6x-higtag at the C-terminus. The resulting expression vector pET-22b(+):*Srldc* was transformed into the *E*. *coli* strain BL21(DE3)-T1^R^, which was grown in 1 L of LB medium containing 100 μg/mL ampicillin at 37°C until the OD 600 reached 0.7. After induction with 1.0 mM 1-thio-β-D-galactopyranoside (IPTG), the culture medium was maintained for a further 20 h at 18°C. The culture was then harvested by centrifugation at 4,000 × *g* for 20 min at 4°C. The cell pellet was resuspended in buffer A (40 mM Tris-HCl, pH 8.0) and disrupted by ultrasonication. The cell debris was removed by centrifugation at 13,500 × *g* for 30 min and the lysate was applied to a Ni-NTA agarose column (Qiagen). After washing with buffer A containing 30 mM imidazole, the bound proteins were eluted with 300 mM imidazole in buffer A. Finally, trace amounts of contaminants were removed by size-exclusion chromatography using a Superdex 200 prep-grade column (320 mL, GE Healthcare) equilibrated with buffer A. All purification experiments were performed at 4°C. SDS-polyacrylamide gel electrophoresis analysis of the purified proteins showed a single polypeptide of 44.0 kDa that corresponded to the estimated molecular weight of the *Sr*LDC monomer. The purified protein was concentrated to 65 mg mL^-1^ in 40 mM Tris-HCl, pH 8.0. Site-directed mutagenesis experiments were performed using the Quick Change site-directed mutagenesis kit (Stratagene). The production and purification of the *Sr*LDC mutants were carried out by the same procedure employed for the wild-type protein. Primers used for cloning and site-directed mutagenesis are listed in S1 Table.

### UV/Vis spectroscopy

The amount of PLP covalently bound to the *Sr*LDC proteins was monitored using UV/Vis absorbance spectroscopy [21,22]. The final concentration of 45.42 μM of *Sr*LDC proteins was used. Spectra of the *Sr*LDC proteins between 300 and 500 nm were recorded on a SHIMADZU UV-VIS Spectrophotometer UV-1800 in 0.1 M potassium phosphate (pH 6.0) buffer.

### Isothermal titration calorimetry

The binding affinity between the *Sr*LDC proteins and PLP was measured using a Nano ITC model (TA Instruments) at 20°C. Protein samples at 100 μM were prepared in 40 mM Tris, pH 8.0. For titrations of *Sr*LDC with PLP, 1.96 μl of 2.5 mM PLP was injected 25 times and each injection was monitored at 200-second intervals. The protein solution in the ITC reaction cell was stirred at 250 RPM. The heat of cofactor dilution into the buffer was subtracted from the reaction heat. Calculated heat area per injection was used for fitting in the independent binding model for calculation of the thermodynamic parameter (*K*_d_).

### LDC activity assay

To measure the lysine decarboxylase (LDC) activity of the *Sr*LDC proteins, 56.78 μM of purified enzyme was added to 200 μl of reaction mixture containing 0.1 M potassium phosphate, pH 6.0, 50 μM l-lysine, and various concentrations of PLP. The reaction mixtures were incubated at 37°C for 30 sec. The reaction was stopped by heating the solution at 90°C for 5 min. After centrifugation at 13,500 × *g* for 5 min, the remaining l-lysine was detected using the lysine oxidase/peroxidase method. Lysine oxidase converts the remaining l-lysine into 6-amino-2-oxohexanoate, NH_3_, and H_2_O_2_, and the H_2_O_2_ is then reduced by peroxidase using 2,2'-azino-bis(3-ethylbenzothiazoline-6-sulphonic acid) (ABTS). After the LDC reaction, equal volume of 2x lysine oxidase/peroxidase solution (0.1 unit ml^-1^ lysine oxidase, 1 unit ml^-1^ peroxidase, and 3.6 mM ABTS in 0.1 M potassium phosphate buffer, pH 8.0) was added to the LDC reaction mixture. The amount of oxidized ABTS was detected by measuring absorbance at 412 nm. To investigate the effect of pH on the LDC activity, the LDC activity assay was carried out at a pH range from 5 to 10. For thermal stability experiments, enzymes were pre-incubated at 37°C and 60°C, and the pre-incubated *Sr*LDC proteins were used in the LDC activity assays. All experiments were performed in triplicate.

### Cadaverine conversion assay

The cadaverine conversion assay was performed using a procedure similar to that described for the LDC activity assay. The conversion mixture of 5 mL contained 0.5 M potassium phosphate buffer (pH 6.0), 0.5 M l-lysine, 4.54 μM of purified enzyme, and various concentrations of PLP. The conversion mixture was then incubated at 37°C for 30 min. Samples were periodically collected and the cadaverine conversion was monitored by measuring the amount of the remaining l-lysine.

### Melting temperature (*T*_m_) measurement

Thermal stability of the *Sr*LDC proteins was determined by measuring melting curves with the Protein thermal shift dye (Applied Biosystems) in a StepOnePlus Real-Time PCR (Thermo Fisher Scientific) according to manufacturer’s instructions. Briefly, 1 μg of protein was mixed with 1x protein thermal shift dye (Applied Biosystems) in 20 μl and signal changes reflecting protein denaturation were monitored by increasing temperature from 25 to 90°C. Melting temperatures were determined from the first derivative curve.

### Crystallization, data collection and structure determination of *Sr*LDC^A225C/T302^

Crystallization of the purified *Sr*LDC^A225C/T302C^ mutant protein was initially performed with commercially available sparse-matrix screens from Rigaku and Molecular Dimensions by using the hanging-drop vapor-diffusion method at 20°C. Each experiment consisted of mixing 1.0 μL of protein solution (65 mg mL^-1^ in 40 mM Tris-HCl, pH 8.0) with 1.0 μL of reservoir solution and equilibrating the drop against 0.5 mL of reservoir solution. The *Sr*LDC^A225C/T302C^ mutant was crystallized in the condition of 1.4 M ammonium sulfate, 0.1 M sodium cacodylate, pH 6.5, and 0.2 M sodium chloride. The crystals were transferred to a cryo-protectant solution composed of the corresponding condition described above and 30% (v/v) glycerol, fished out with a loop larger than the crystals, and flash-frozen by immersion in liquid nitrogen. All data were collected at the 7A beamline of the Pohang Accelerator Laboratory (PAL, Pohang, Korea), using a Quantum 270 CCD detector (ADSC, USA). The *Sr*LDC^A225C/T302C^ crystals diffracted to 1.8 Å resolutions. All data were indexed, integrated, and scaled using the HKL-2000 software package [23]. The *Sr*LDC^A225C/T302C^ crystals belonged to the space group *P*2_1_2_1_2_1_ with unit cell dimensions of *a* = 55.91 Å, *b* = 122.85 Å, *c* = 136.85 Å, *α* = *β* = *γ* = 90.0°. With two molecules of *Sr*LDC^A225C/T302C^ per asymmetric unit, the crystal volume per unit protein mass was 2.7 Å^3^ Da^-1^, indicating that the solvent content was 54.0% [24]. The structure of *Sr*LDC^A225C/T302C^ mutant was determined by molecular replacement with CCP4 version of MOLREP [25] using the structure of refined *Sr*LDC as a model. Model building was performed manually using the program WinCoot [26] and structure refinement was performed with CCP4 refmac5 [27]. Data collection and refinement statistics are summarized in Table 1. The structure of *Sr*LDC^A225C/T302C^ mutant was deposited in the Protein Data Bank with PDB codes of 5GJO.

**Table 1:** Data collection and refinement statistics of *Sr*LDC^A225C/T302C^.

	*Sr*LDC^A225C/T302C^
	+ PLP
**PDB code**	5GJO
**Data collection**	
Space group	*P*2_1_2_1_2_1_
Cell dimensions	
*a*, *b*, *c* (Å)	55.9, 122.9, 136.9
α, β, γ (°)	90.0, 90.0, 90.0
Resolution (Å)	50.0–1.8 (1.83–1.8)*
*R*_sym_ or *R*_merge_	7.7 (29.8)
*I* / σ*I*	48.8 (7.5)
Completeness (%)	99.3 (98.3)
Redundancy	11.5 (9.0)
**Refinement**	
Resolution (Å)	50.0–1.8
No. reflections	83265
*R*_work_ / *R*_free_	16.5/19.9
No. atoms	6842
Protein	5960
PLP	32
Glycerol	12
Na^+^	4
SO_4_^2-^	30
Water	804
*B*-factors	22.5
Protein	21.6
PLP	30.7
Glycerol	22.2
Na^+^	47.5
SO_4_^2-^	44.8
Water	32.4
B from Wilson plot (Å^2^)	18.2
R.m.s. deviations	
Bond lengths (Å)	0.019
Bond angles (°)	1.982

* The numbers in parentheses are statistics from the highest resolution shell.

## Results

### Strategy for the introduction of disulfide bond in *Sr*LDC

**Fig 1:**
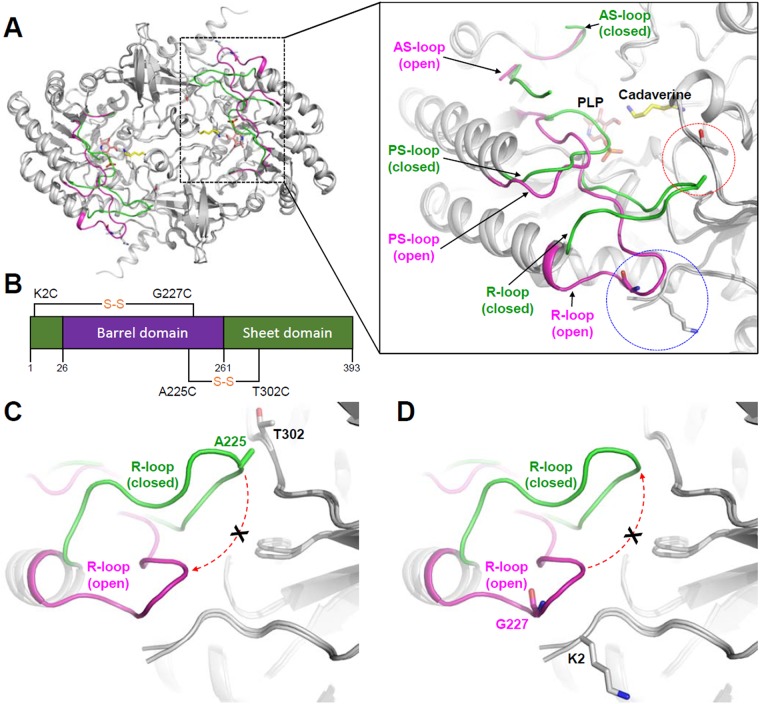
Rationale of introduction of an artificial disulfide bond in *Sr*LDC. (**A**) Open/closed conformational changes at the PLP binding site of *Sr*LDC. The open and the closed conformations of *Sr*LDC are superimposed and shown as a cartoon diagram with green and magenta colors, respectively. The right side figure is an amplification of the dotted-box of the left figure. The AS-loop, PS-loop and R-loop of the open and the closed conformations of *Sr*LDC are labeled. The bound PLP and cadaverine are shown as stick models and labeled. The target residues for the introduction of a disulfide bond are indicated with dotted-circle. (**B**) Schematic of the introduced disulfide bonds in *Sr*LDC. The sheet domain and barrel domain are presented with green and purple colors, respectively. The residues replaced by cysteines to introduce a disulfide bond are presented with black line and labeled. (**C**) (**D**) A rationale of introduction of a disulfide bond. The open and the closed conformations of *Sr*LDC are superimposed and shown as a cartoon diagram. The R-loop of the open and closed conformation of *Sr*LDC are presented with cartoon diagrams with the same color scheme with (**A**). The residues selected to generate the *Sr*LDC^A225C/T302C^ mutant (**C**) and the *Sr*LDC^K2C/G227C^ mutant (**D**) are shown as stick models and labeled.

In the previous study, we determined the crystal structures of *Sr*LDC in several different forms and revealed that the protein has a highly flexible PLP site [20]. We also reported that, due to the flexible PLP binding site, the protein undergoes an open/closed conformational change at the PLP binding site depending on the PLP binding (Fig 1A). Especially, two loops located in the vicinity of the PLP binding site, the PLP stabilization loop (PS-loop) and the regulatory loop (R-loop), undergoes a significant structural movement depending on the PLP binding (Fig 1A). In the open conformation of *Sr*LDC, the PS-loop and the R-loop move away from the PLP binding site by a distance of 5.0 and 12.2 Å, respectively, compared with the closed form of the protein. The open/closed conformation change by the highly flexible PLP binding site causes a low PLP affinity for the enzyme and consequently requires continuous supplement of PLP in the production of cadaverine. Most of other PLP-dependent enzymes contain highly stable active site and they hold its cofactor strongly with high affinities for PLP [28–34]. These structural and biochemical studies indicate that the high stability at the PLP binding site contributes to the high affinity for PLP. In order to develop a *Sr*LDC mutant with an enhanced PLP affinity and thus make the production of cadaverine using the enzyme be a cost-effective process, we performed the structure-based protein engineering. The rationale of the protein engineering is to increase the stability of the PLP binding site and consequently maintain the site in the closed conformation. Based on the structural conformation of the closed form of *Sr*LDC, we decided to introduce an internal disulfide bond between the R-loop and its neighboring region. We selected two target residues, Ala225 and Thr302 located at the R-loop and its neighboring region, respectively, and generated the *Sr*LDC^A225C/T302C^ mutant by replacing these residues to cysteine (Fig 1B and 1C). These two residues contribute to the stabilization of the R-loop in the closed conformation by forming a hydrogen bond and are considered to be located in a suitable distance to form a disulfide bond each other when mutated to cysteine. We expect that the closed conformation of the R-loop by the introduced disulfide bond might subsequently cause the PS-loop to be maintained in the closed conformation (Fig 1C). This spatial reconstitution by introduction of the artificial disulfide bond might consequently make the PLP binding site be maintained in the closed conformation and increase the affinity for PLP. To prepare the negative control, we also designed a mutant that maintains the PLP binding site in the open conformation (Fig 1D). In the open conformation of *Sr*LDC, the R-loop is stabilized by interaction with the N-terminal region, and we selected two residues, Gly227 located at the R-loop and Lys2 at the N-terminal region, and generated the *Sr*LDC^K2C/G227C^ mutant by replacing these residues to cysteine (Fig 1B and 1D). We expect that the PLP binding site of the mutant to be maintained in the open conformation by the introduced disulfide bond and exhibits much lower PLP affinity than the wild-type.

### Enhanced PLP affinity of the *Sr*LDC^A225C/T302C^ mutant

**Fig 2:**
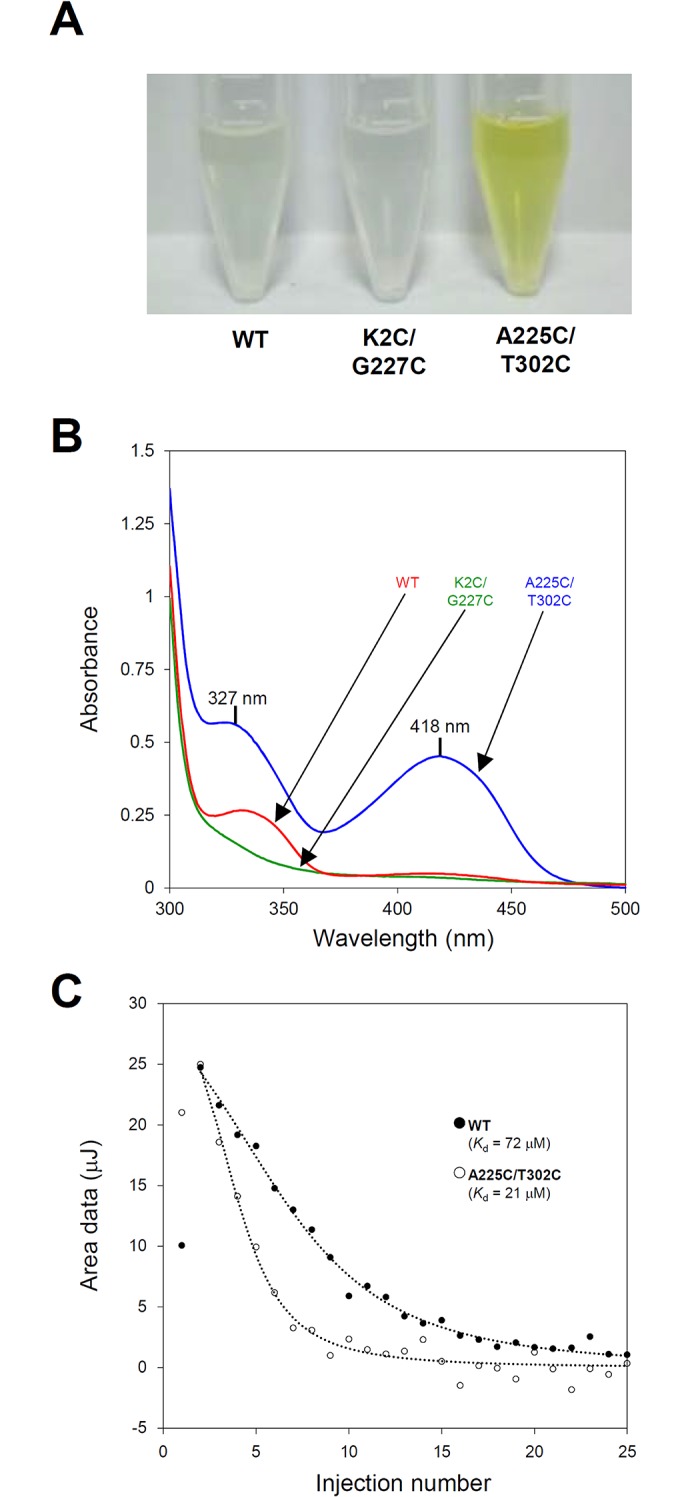
Enhanced PLP affinity in the *Sr*LDC^A225C/T302C^ mutant. (**A**) Purified recombinant *Sr*LDC proteins. The purified *Sr*LDC^WT^, *Sr*LDC^K2C/G227C^, and *Sr*LDC^A225C/T302C^ proteins with concentration of 1 mg/ml are shown. (**B**) UV/vis spectra of recombinant *Sr*LDC proteins. The UV/Vis absorption spectra of recombinant *Sr*LDC proteins are shown and labeled appropriately. The band peaks of 327 nm and 418 nm observed in the *Sr*LDC^A225C/T302C^ mutant are indicated. (**C**) Isothermal titration calorimetry of *Sr*LDC^WT^ and *Sr*LDC^A225C/T302C^ mutant. *K*_d_ values of *Sr*LDC^WT^ and the *Sr*LDC^A225C/T302C^ mutant are displayed.

We first purified the three recombinant *Sr*LDC proteins, *Sr*LDC^WT^, *Sr*LDC^A225C/T302C^, and *Sr*LDC^K2C/G227C^. Surprisingly, the *Sr*LDC^A225C/T302C^ mutant showed a clear yellow color compared with *Sr*LDC^WT^ and the *Sr*LDC^K2C/G227C^ mutant, indicating that the *Sr*LDC^A225C/T302C^ mutant tightly holds the PLP cofactor (Fig 2A). To compare the amount of PLP bound to the three *Sr*LDC proteins, we then performed the UV/Vis absorption spectra scanning from 300 to 500 nm. The *Sr*LDC^A225C/T302C^ mutant showed absorption peaks at 327 and 418 nm, a characteristic of internal aldimine formed between PLP and enzyme (Fig 2B). On the other hand, *Sr*LDC^WT^ and the *Sr*LDC^K2C/G227C^ mutant showed no detectable absorption spectra (Fig 2B). These results indicate that the *Sr*LDC^A225C/T302C^ mutant retained much more PLP in its binding site than *Sr*LDC^WT^ or *Sr*LDC^K2C/G227C^ mutant during the expression and purification procedures. We also performed the isothermal titration calorimetry experiments and compared the *K*_d_ value of the *Sr*LDC^A225C/T302C^ mutant with that of *Sr*LDC^WT^. *Sr*LDC^WT^ and the *Sr*LDC^A225C/T302C^ mutant showed *K*_d_ values of 72 and 21 μM, respectively, indicating that the *K*_d_ value of the *Sr*LDC^A225C/T302C^ mutant was increased in 3.4 fold compared with that of *Sr*LDC^WT^ (Fig 2C). These results suggest that introduction of the disulfide bond indeed increased the PLP affinity of the *Sr*LDC^A225C/T302C^ mutant.

### Enhanced LDC activity of the *Sr*LDC^A225C/T302C^ mutant

**Fig 3:**
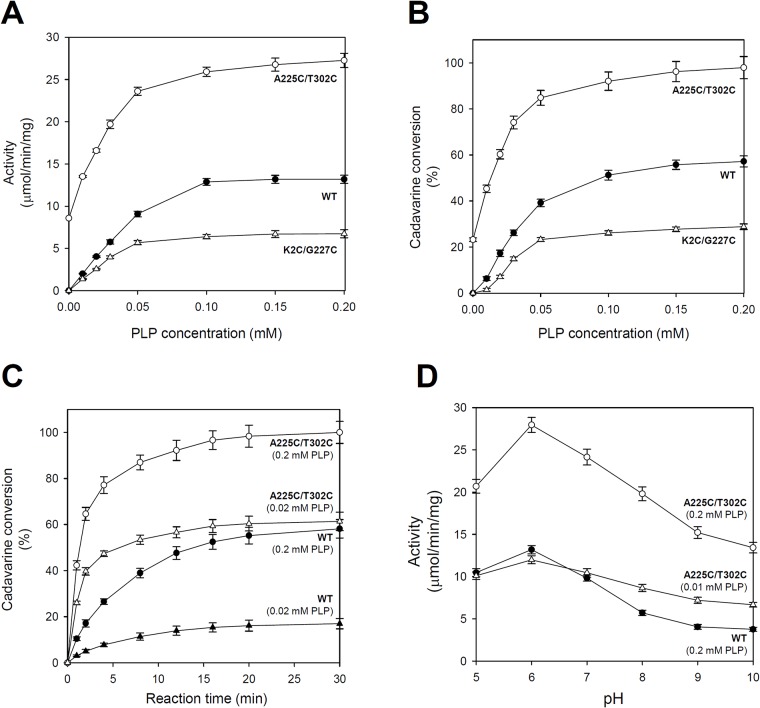
Comparison of LDC activity between *Sr*LDC proteins (**A**) Comparison of LDC activities of the *Sr*LDC proteins. LDC activities of *Sr*LDC^WT^, *Sr*LDC^K2C/G227C^, and *Sr*LDC^A225C/T302C^ are measured at various concentrations of PLP. (**B**) Comparison of cadaverine conversion of the *Sr*LDC proteins. Cadaverine conversion of *Sr*LDC^WT^, *Sr*LDC^K2C/G227C^, and *Sr*LDC^A225C/T302C^ are measured at various concentrations of PLP. (**C**) Comparison of time-course cadaverine conversion between *Sr*LDC^WT^ and *Sr*LDC^A225C/T302C^. Time-course cadaverine conversion of *Sr*LDC^WT^ and the *Sr*LDC^A225C/T302C^ mutant are measured under PLP concentrations of 0.02 and 0.2 mM. (**D**) Comparison of resistance to pH between *Sr*LDC^WT^ and *Sr*LDC^A225C/T302C^. The LDC activity of *Sr*LDC^WT^ is measured at pH from 5 to 10 under 0.2 mM PLP. The LDC activities of the *Sr*LDC^A225C/T302C^ mutant are also measured at the same pH range under 0.01 and 0.2 mM PLP.

To investigate how the enhanced PLP affinity influences on the LDC activity, we compared the LDC activity of the *Sr*LDC^A225C/T302C^ mutant with those of *Sr*LDC^WT^ and the *Sr*LDC^K2C/G227C^ mutant under various concentrations of PLP. Surprisingly, the *Sr*LDC^A225C/T302C^ mutant showed a two-fold higher activity when compared to *Sr*LDC^WT^ with supplement of 0.2 mM of PLP, and the *Sr*LDC^K2C/G227C^ mutant showed only half of the *Sr*LDC^WT^ activity at the same concentration of PLP (Fig 3A). More dramatic differences were observed with low or no PLP supplement. Although *Sr*LDC^WT^ and the *Sr*LDC^K2C/G227C^ mutant showed no detectable activity without PLP supplement, the *Sr*LDC^A225C/T302C^ mutant exhibited LDC activity corresponding to 65% of *Sr*LDC^WT^ activity in the presence of 0.2 mM PLP (Fig 3A). Moreover, the activity of *Sr*LDC^A225C/T302C^ mutant in the presence of 0.01 mM PLP was almost identical to that of *Sr*LDC^WT^ in the presence of 0.2 mM PLP (Fig 3A). In order to investigate conversion to cadaverine in a condition that is close to the real cadaverine production, we measured the conversion rate to cadaverine by addition of 0.5 M of lysine. In the presence of 0.2 mM PLP, the *Sr*LDC^A225C/T302C^ mutant showed almost 100% cadaverine conversion, whereas *Sr*LDC^WT^ and the *Sr*LDC^K2C/G227C^ did only 60% and 30%, respectively (Fig 3B). Similar to the activity comparison described above, more dramatic differences were observed with low or no PLP supplement. The *Sr*LDC^A225C/T302C^ mutant converted 25% of lysine into cadaverine even without PLP supplement, whereas both *Sr*LDC^WT^ and the *Sr*LDC^K2C/G227C^ mutant showed no detectable cadaverine conversion (Fig 3B). Moreover, the *Sr*LDC^A225C/T302C^ mutant in the presence of only 0.02 mM of PLP converted more lysine into cadaverine than *Sr*LDC^WT^ did in the presence of 0.2 mM of PLP (Fig 3B). Measuring the time course of cadaverine conversion showed that the difference in cadaverine conversion between *Sr*LDC^WT^ and *Sr*LDC^A225C/T302C^ mutant was even more conspicuous at the initial phase of the conversion period (Fig 3C). With 0.02 mM of PLP, the *Sr*LDC^A225C/T302C^ mutant exhibited even higher cadaverine conversion than *Sr*LDC^WT^ with 0.2 mM of PLP. We suspect that the enhanced LDC activity and cadaverine conversion of mutant *Sr*LDC^A225C/T302C^, in particular with low or no PLP supplement, resulted from an enhanced stabilization of the PLP binding site by the disulfide bond-mediated spatial reconstitution.

In addition, we also investigate whether the *Sr*LDC^A225C/T302C^ mutant shows resistance against pH increase. LDCs are known to have a low optimum pH and show reduced activity when the pH is increased. Because one proton ion is consumed by each decarboxylation reaction, leading to an increase in pH, LDCs tend to lose their activity as the reaction proceeds. We measured the LDC activity of the *Sr*LDC^A225C/T302C^ mutant at pH ranging from 5 to 10 and compared with that of *Sr*LDC^WT^. As we expected, both the *Sr*LDC^A225C/T302C^ mutant and *Sr*LDC^WT^ exhibited the highest activity at pH 6.0, and the activity of both enzymes gradually decreased while pH increased (Fig 3D). However, it is important to note that the *Sr*LDC^A225C/T302C^ mutant showed a higher activity than *Sr*LDC^WT^ did throughout the whole pH range, and the difference in activity between the two enzymes was even more pronounced at a higher pH (Fig 3D). When the LDC activity of the mutant was measured in the presence of 0.01 mM PLP, the resistance of the *Sr*LDC^A225C/T302C^ mutant against pH increase was more apparent than that shown in the presence of 0.2 mM PLP. At pH 10, the activity of the mutant enzyme in the presence of only 0.01 mM PLP was up to two-fold higher than that of *Sr*LDC^WT^ in the presence of 0.2 mM PLP (Fig 3D). These results indicate that the *Sr*LDC^A225C/T302C^ mutant, compared with *Sr*LDC^WT^, maintains its relatively high activity even when pH increases as a result from prolonged reactions.

### Enhanced thermal stability of the *Sr*LDC^A225C/T302C^ mutant

**Fig 4:**
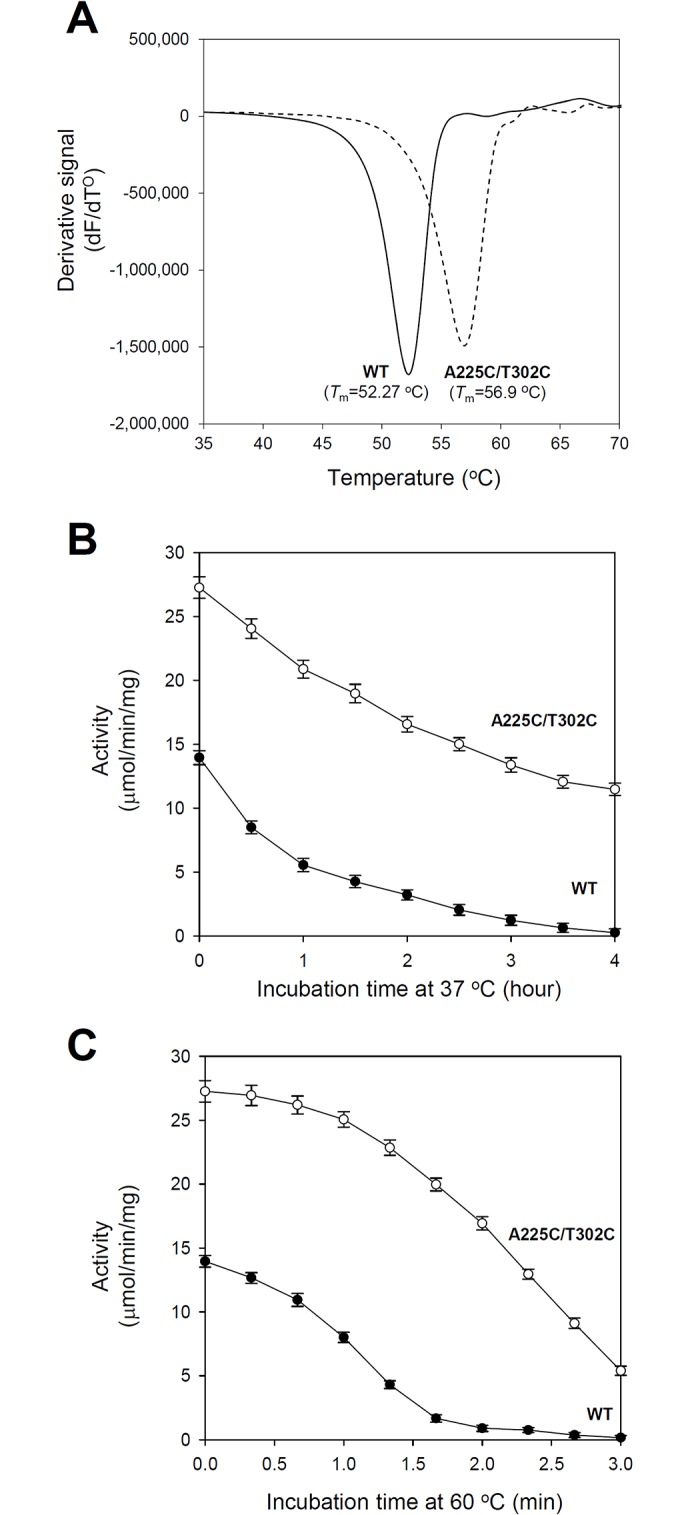
Comparison of resistance to temperature between *Sr*LDC^WT^ and *Sr*LDC^A225C/T302C^. (**A**) Thermal stability measurement of *Sr*LDC^WT^ and *Sr*LDC^A225C/T302C^. The *T*_m_ values of *Sr*LDC^WT^ and the *Sr*LDC^A225C/T302C^ mutant are displayed. (**B**) (**C**) Comparison of thermal stability between *Sr*LDC^WT^ and *Sr*LDC^A225C/T302C^. The LDC activities of *Sr*LDC^WT^ and the *Sr*LDC^A225C/T302C^ mutant are measured using proteins pre-incubated at 37°C (**B**) and 60°C (**C**).

In general, introduction of internal disulfide bonds in proteins results in an increase in thermostability of the engineered proteins [35–37]. Although we introduced the internal disulfide bond to increase the stability of the active site, we expected an enhanced thermostability of the *Sr*LDC^A225C/T302C^ mutant. We then measured the melting temperature (*T*_m_) of the mutant enzyme and compared with that of *Sr*LDC^WT^. Interestingly, the *Sr*LDC^A225C/T302C^ mutant showed a *T*_m_ of 56.9°C, which is higher than the *T*_m_ of 52.27°C observed for *Sr*LDC^WT^ (Fig 4A). Next, we investigated how prolonged incubation at 37°C affected the activity of the proteins. Only 50% of *Sr*LDC^WT^ activity remained after incubation for one hour, and an almost complete loss of activity was observed after four hours of incubation (Fig 4B). In contrast, the activity of the *Sr*LDC^A225C/T302C^ mutant remained relatively high, with almost half of it present even after four hours of incubation (Fig 4B). Even after four hours of incubation, the *Sr*LDC^A225C/T302C^ mutant showed almost identical activity to that of *Sr*LDC^WT^ without incubation (Fig 4B). We also measured the activity of the proteins after incubation at 60°C. *Sr*LDC^WT^ showed an immediate decrease in activity during incubation at this temperature, and an almost complete loss of activity after an incubation of two min (Fig 4C). In contrast, the *Sr*LDC^A225C/T302C^ mutant retained a relatively high activity up to two min of incubation at 60°C, and still showed activity after three min of incubation (Fig 4C). These results indicate that the disulfide bond-mediated spatial reconstitution resulted in not only enhanced stability at the active site but also in increased thermostability of the enzyme.

### Crystal structure of the *Sr*LDC^A225C/T302C^ mutant

**Fig 5:**
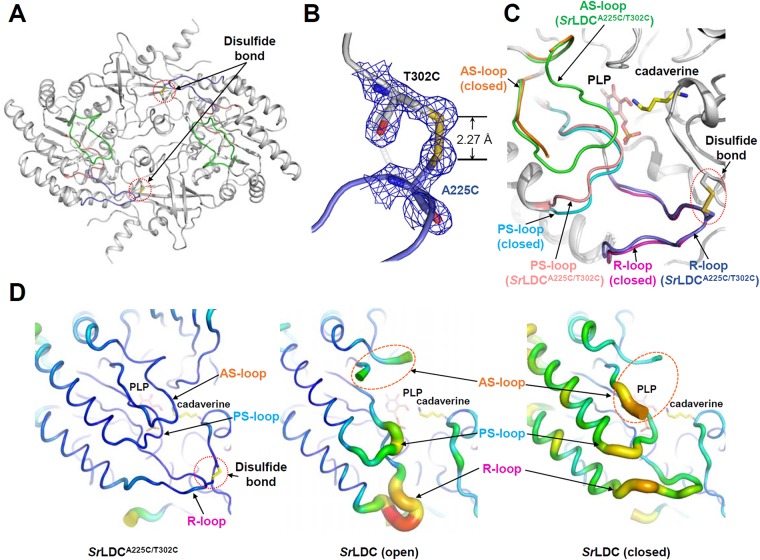
Crystal structure of the *Sr*LDC^A225C/T302C^ mutant. (**A**) Dimeric structure of the *Sr*LDC^A225C/T302C^ mutant. The AS-loop, PS-loop and the R-loop of the *Sr*LDC^A225C/T302C^ mutant are colored green, salmon, and light-blue, respectively. The disulfide bond formed in both monomers are indicated are shown as stick model and labeled. (**B**) Electron density map of the engineered disulfide bond region. The Fo-Fc map of the engineered disulfide bond region is contoured at 3.0 σ. The R-loop region is presented with a light-blue color and the mutated residues are shown as stick models with labels. (**C**) Superimposition of the *Sr*LDC^A225C/T302C^ structure with the closed form of *Sr*LDC. The AS-loop, the PS-loop, and the R-loop of the *Sr*LDC^A225C/T302C^ mutant are distinguished with colors of green, salmon, and light-blue, respectively, and those of the closed form of *Sr*LDC are with colors of orange, cyan, and magenta, respectively. (**D**) B-factor presentation of the *Sr*LDC^A225C/T302C^ mutant and *Sr*LDC in apo and complex with PLP/cadaverine. The AS-loop, the PS-loop, and the R-loop are labeled.

To structurally confirm the enhanced stability of the PLP binding site in mutant *Sr*LDC^A225C/T302C^ is derived from the introduced disulfide bond, we determined its crystal structure at 1.8 Å resolution (Fig 5A and Table 1). As expected, a disulfide bond was observed between A225C and T302C, and the PS-loop and the R-loop of the mutant showed the closed conformation (Fig 5B and 5C). Moreover, When we compared the B-factors of the PS-loop and the R-loop in *Sr*LDC^A225C/T302C^ with those in the wild-type *Sr*LDC, B-factors for the PS-loop and the R-loop of the *Sr*LDC^A225C/T302C^ mutant were much lower than those of both the open and closed forms of *Sr*LDC^WT^ (Fig 5D), indicating that the stability of the loop regions are significantly enhanced by the engineered disulfide bond. Interestingly, the active site loop (AS-loop), that are known to be highly disordered in both the open and closed forms of *Sr*LDC^WT^, showed a clear electron density map in the *Sr*LDC^A225C/T302C^ mutant (S1 Fig). The loop is stabilized by extensive hydrogen bonds with the PS-loop (S2 Fig). The AS-loop seems to contribute to the formation of the substrate binding site. In particular, the Val146 residue in the AS-loop is positioned in the vicinity of the substrate and seems to be involved in the stabilization of the hydrocarbon part of the lysine substrate (S3 Fig). This observation indicates that the engineered disulfide bond also enhanced the substrate binding affinity. Interestingly, we observed the clear electron density for PLP cofactor even without addition of PLP in the crystallization solution, indicating that the *Sr*LDC^A225C/T302C^ mutant retained the PLP cofactor during purification and crystallization procedures (Fig 5C). Taken together, the engineered disulfide bond seems to result in the maintenance of the R-loop in the closed conformation, and in turn leads to the PS-loop to be retained in the closed conformation as well, which consequently affects the stability of the AS-loop.

## Discussion

Enhanced stability of enzymes with industrial applications may improve production yield and lead to expanded operational environments such as temperature and pH. The introduction of disulfide bonds has been used as a powerful engineering tool to increase protein stability [35,36,38]. Recent studies have also shown that disulfide bond engineering can be used in a wide range of applications such as kinetic stability and functional modification of proteins [39–41]. However, not all engineered disulfide bonds produce an improved enzyme due to the difficulty in predicting the conformation and thermodynamics of an engineered disulfide bond. Our successful protein engineering on *Sr*LDC has two unique features compared with the previously reported works. First, we used disulfide bond-mediated spatial reconstitution at the cofactor binding site to increase cofactor affinity rather than increase the stability of protein folding itself. Second, we achieved the equivalent of killing two birds with a single stone; one engineered disulfide bond enhanced both the enzymatic activity and the resistance to pH and temperature of the target protein. In many cases, engineered disulfide bonds lead to the increase of either enzymatic activity or enzyme stability [42]. Extensive structural analysis seem to enable this protein engineering to be successful.

Here, it is worth to notice that appropriate flexibility at the active site is important for maximum enzymatic activity. As described above, the *Sr*LDC^A225C/T302C^ mutant shows a stabilized AS-loop and forms the closed substrate binding site. Based on these findings, we introduced an additional disulfide bond between the AS-loop and the PS-loop in the *Sr*LDC^A225C/T302C^ mutant and generated *Sr*LDC^K143C/L185C/A225C/T302C^ mutant (S4 Fig). We anticipate that the more stable active site results in the improved LDC activity of the mutant. As expected, the purified quadruple mutant showed a yellow color similar to the *Sr*LDC^A225C/T302C^ mutant. However, its enzymatic activity was only 65% even compared with that of *Sr*LDC^WT^ (S4 Fig). We speculate that the low activity of the quadruple mutant is caused by a high rigidity of the substrate biding site. Based on these results, we suggest that the maintenance of an appropriate flexibility at the active site is one of the important factors to be considered when we design a structure-based protein engineering.

PLP is a cofactor required in a variety of enzyme reactions such as transamination and decarboxylation [43]. Especially, enzymes involved in the biosynthesis of amino acids and their derivatives utilize PLP as an essential cofactor. For this reason, PLP has been one of the critical control factors for the production of industrially important bioproducts and many attempts have been also made to reduce the cost by a continuous supplement of PLP. As a platform technology, this approach could be used for the development of highly efficient PLP-dependent enzymes that would allow more cost-effective production of valuable bioproducts.

## Supporting Information

S1 Fig Electron density map of the AS-loop in the *Sr*LDC^A225C/T302C^ structure. The AS-loop region (Ile137-Gly153) of the *Sr*LDC^A225C/T302C^ structure is shown as a stick model and the Fo-Fc map is contoured at 3.0 σ.
(PPTX)

S2 Fig Stereoview of Stabilization of the AS-loop, the PS-loop, and the R-loop of the *Sr*LDC^A225C/T302C^ mutant. The AS-loop, the PS-loop, and the R-loop of the *Sr*LDC^A225C/T302C^ mutant are presented with green, salmon, and light-blue color, respectively. Residues involved in the stabilization of the loops are presented with stick and line models, and labeled.
(PPTX)

S3 Fig Stereoview of involvement of V146 in the substrate binding. The AS-loop and PS-loop in the *Sr*LDC^A225C/T302C^ mutant are colored green and salmon, respectively. The residues involved in the substrate binding are shown as a stick model and labeled and Val146 is distinguished with green color. The cadaverine molecule and the PLP cofactor are shown as stick models with yellow and salmon colors, respectively.
(PPTX)

S4 Fig The *Sr*LDC^K143C/L185C/A225C/T302C^ mutant. (**A**) Rationale of introducing an additional disulfide bond to generate the *Sr*LDC^K143C/L185C/A225C/T302C^ mutant. The *Sr*LDC^A225C/T302C^ structure is shown as a cartoon diagram. The AS-loop, PS-loop and the R-loop are colored green, salmon, and light-blue, respectively. The disulfide bond formed between A225C and T302C is shown as stick model. Residues K143 and L185 replaced by cysteine residues to form an additional disulfide bond are shown as stick models and labeled. (**B**) Activity of the *Sr*LDC^K143C/L185C/A225C/T302C^ mutant.
(PPTX)

S1 Table Primers used for cloning and site-directed mutagenesis of *Sr*LDC. (PPTX)
