# Supplementary material for: Lysine Decarboxylase with an Enhanced Affinity for Pyridoxal 5-Phosphate by Disulfide Bond-Mediated Spatial Reconstitution
Source: PLoS One. 2017 Jan 17;12(1):e0170163. doi: 10.1371/journal.pone.0170163 (PMC5240995; doi:10.1371/journal.pone.0170163)
Supplement: S1 Fig — The AS-loop region (Ile137-Gly153) of the SrLDCA225C/T302C structure is shown as a stick model and the Fo-Fc map is contoured at 3.0 σ. (PPTX) [file pone.0170163.s001.pptx]

## Slide 1
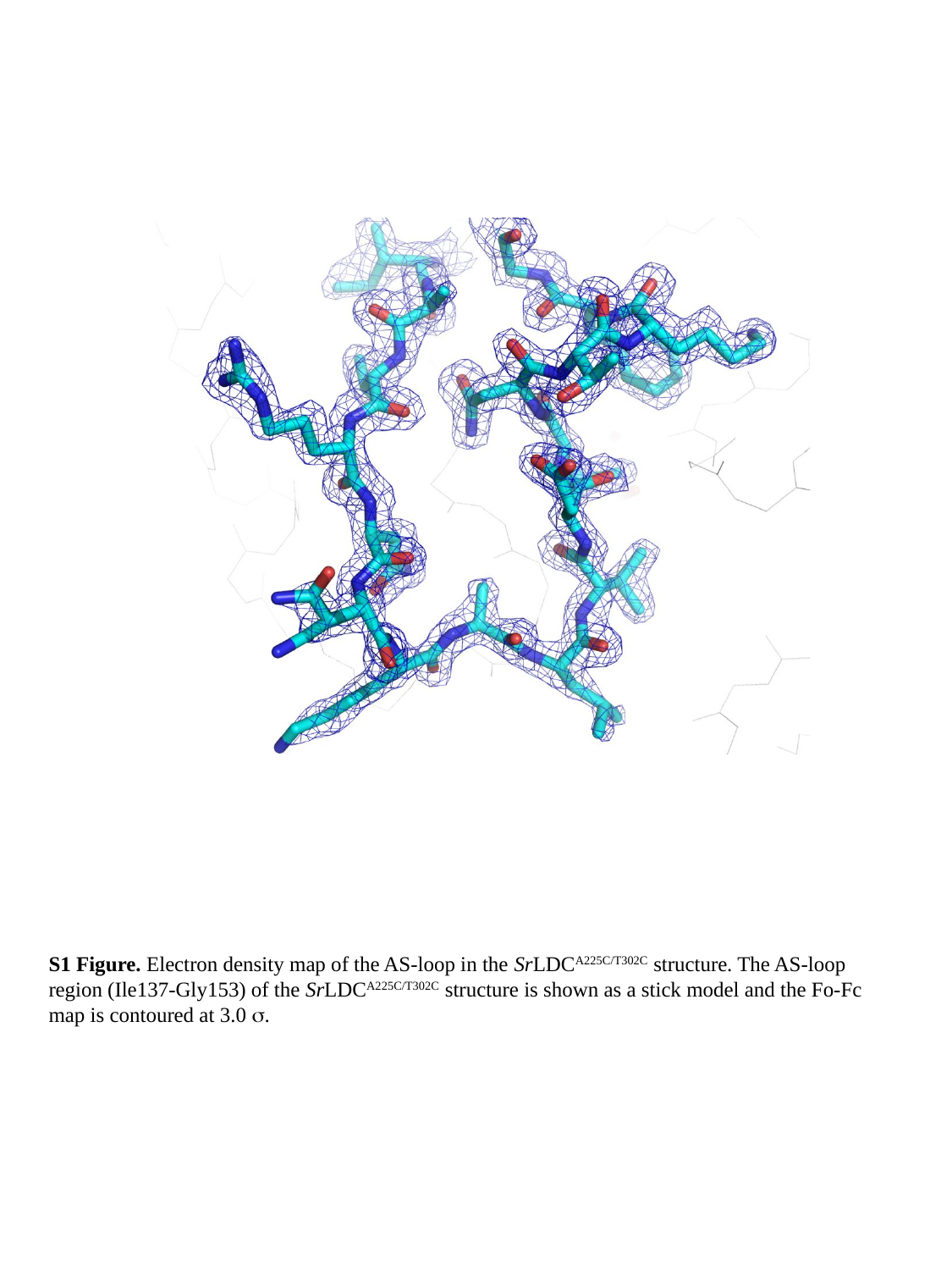

S1 Figure. Electron density map of the AS-loop in the SrLDCA225C/T302C structure. The AS-loop region (Ile137-Gly153) of the SrLDCA225C/T302C structure is shown as a stick model and the Fo-Fc map is contoured at 3.0 s.
