# Supplementary material for: Lysine Decarboxylase with an Enhanced Affinity for Pyridoxal 5-Phosphate by Disulfide Bond-Mediated Spatial Reconstitution
Source: PLoS One. 2017 Jan 17;12(1):e0170163. doi: 10.1371/journal.pone.0170163 (PMC5240995; doi:10.1371/journal.pone.0170163)
Supplement: S2 Fig — The AS-loop, the PS-loop, and the R-loop of the SrLDCA225C/T302C mutant are presented with green, salmon, and light-blue color, respectively. Residues involved in the stabilization of the loops are presented with stick and line models, and labeled. (PPTX) [file pone.0170163.s002.pptx]

## Slide 1
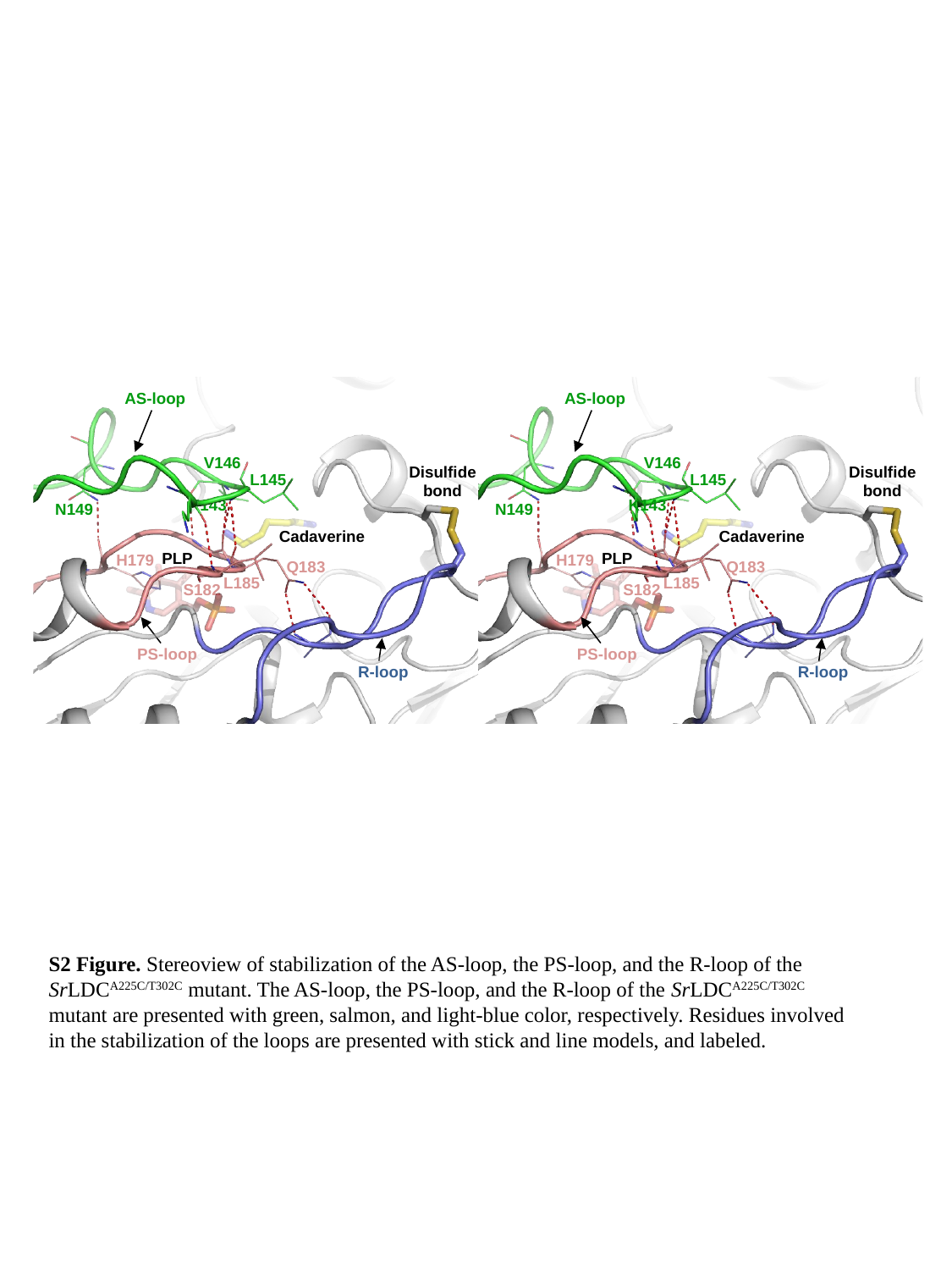

AS-loop
V146
Disulfide
bond
L145
K143
N149
Cadaverine
PLP
H179
Q183
L185
S182
PS-loop
R-loop
AS-loop
V146
Disulfide
bond
L145
K143
N149
Cadaverine
PLP
H179
Q183
L185
S182
PS-loop
R-loop
S2 Figure. Stereoview of stabilization of the AS-loop, the PS-loop, and the R-loop of the SrLDCA225C/T302C mutant. The AS-loop, the PS-loop, and the R-loop of the SrLDCA225C/T302C mutant are presented with green, salmon, and light-blue color, respectively. Residues involved in the stabilization of the loops are presented with stick and line models, and labeled.
