# Supplementary material for: Lysine Decarboxylase with an Enhanced Affinity for Pyridoxal 5-Phosphate by Disulfide Bond-Mediated Spatial Reconstitution
Source: PLoS One. 2017 Jan 17;12(1):e0170163. doi: 10.1371/journal.pone.0170163 (PMC5240995; doi:10.1371/journal.pone.0170163)
Supplement: S3 Fig — The AS-loop and PS-loop in the SrLDCA225C/T302C mutant are colored green and salmon, respectively. The residues involved in the substrate binding are shown as a stick model and labeled and Val146 is distinguished with green color. The cadaverine molecule and the PLP cofactor are shown as stick models with yellow and salmon colors, respectively. (PPTX) [file pone.0170163.s003.pptx]

## Slide 1
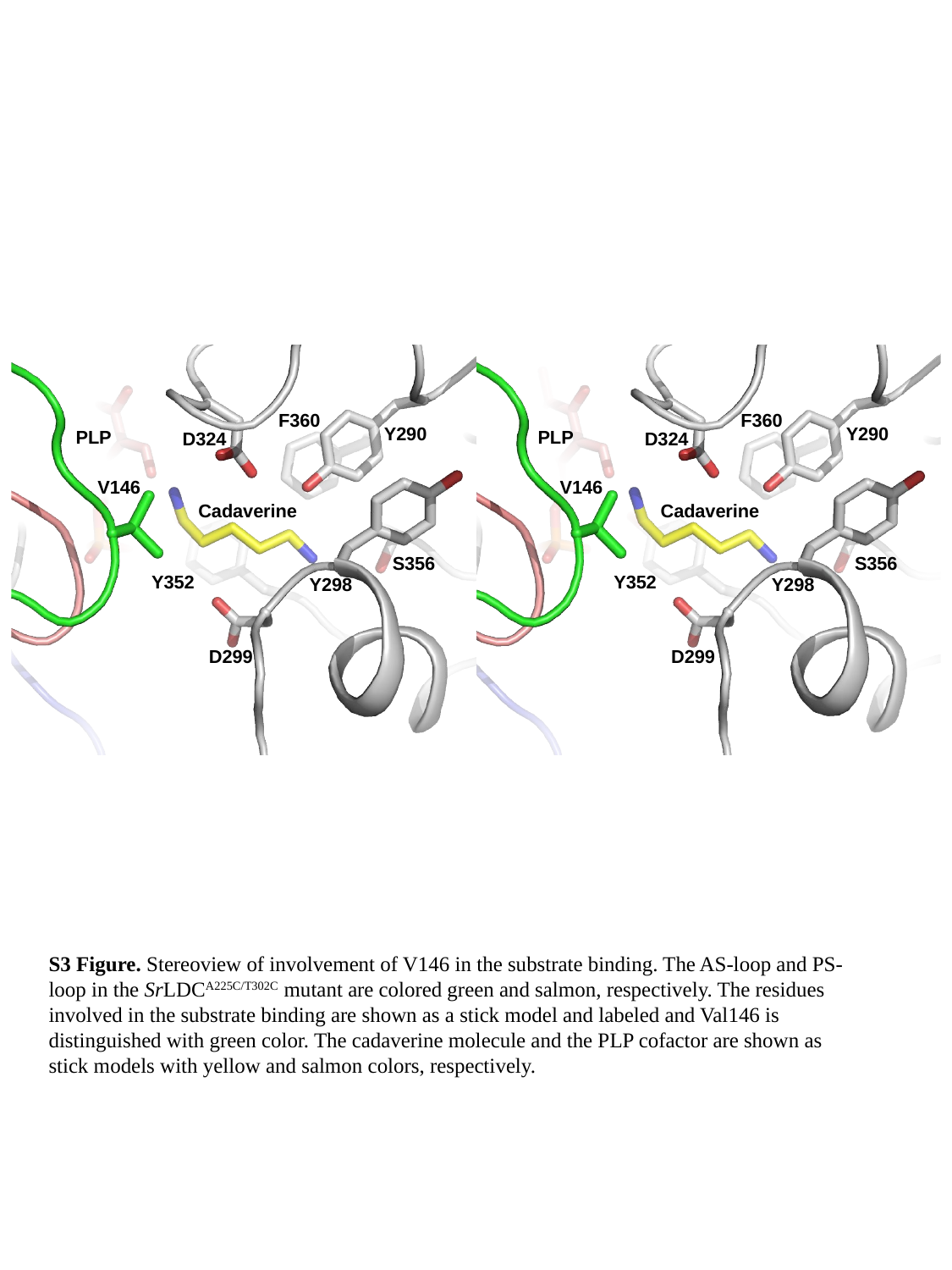

F360
Y290
PLP
D324
V146
Cadaverine
S356
Y352
Y298
D299
F360
Y290
PLP
D324
V146
Cadaverine
S356
Y352
Y298
D299
S3 Figure. Stereoview of involvement of V146 in the substrate binding. The AS-loop and PS-loop in the SrLDCA225C/T302C mutant are colored green and salmon, respectively. The residues involved in the substrate binding are shown as a stick model and labeled and Val146 is distinguished with green color. The cadaverine molecule and the PLP cofactor are shown as stick models with yellow and salmon colors, respectively.
