# Supplementary material for: Lysine Decarboxylase with an Enhanced Affinity for Pyridoxal 5-Phosphate by Disulfide Bond-Mediated Spatial Reconstitution
Source: PLoS One. 2017 Jan 17;12(1):e0170163. doi: 10.1371/journal.pone.0170163 (PMC5240995; doi:10.1371/journal.pone.0170163)
Supplement: S4 Fig — (A) Rationale of introducing an additional disulfide bond to generate the SrLDCK143C/L185C/A225C/T302C mutant. The SrLDCA225C/T302C structure is shown as a cartoon diagram. The AS-loop, PS-loop and the R-loop are colored green, salmon, and light-blue, respectively. The disulfide bond formed between A225C and T302C is shown as stick model. Residues K143 and L185 replaced by cysteine residues to form an additional disulfide bond are shown as stick models and labeled. (B) Activity of the SrLDCK143C/L185C/A225C/T302C mutant. (PPTX) [file pone.0170163.s004.pptx]

## Slide 1
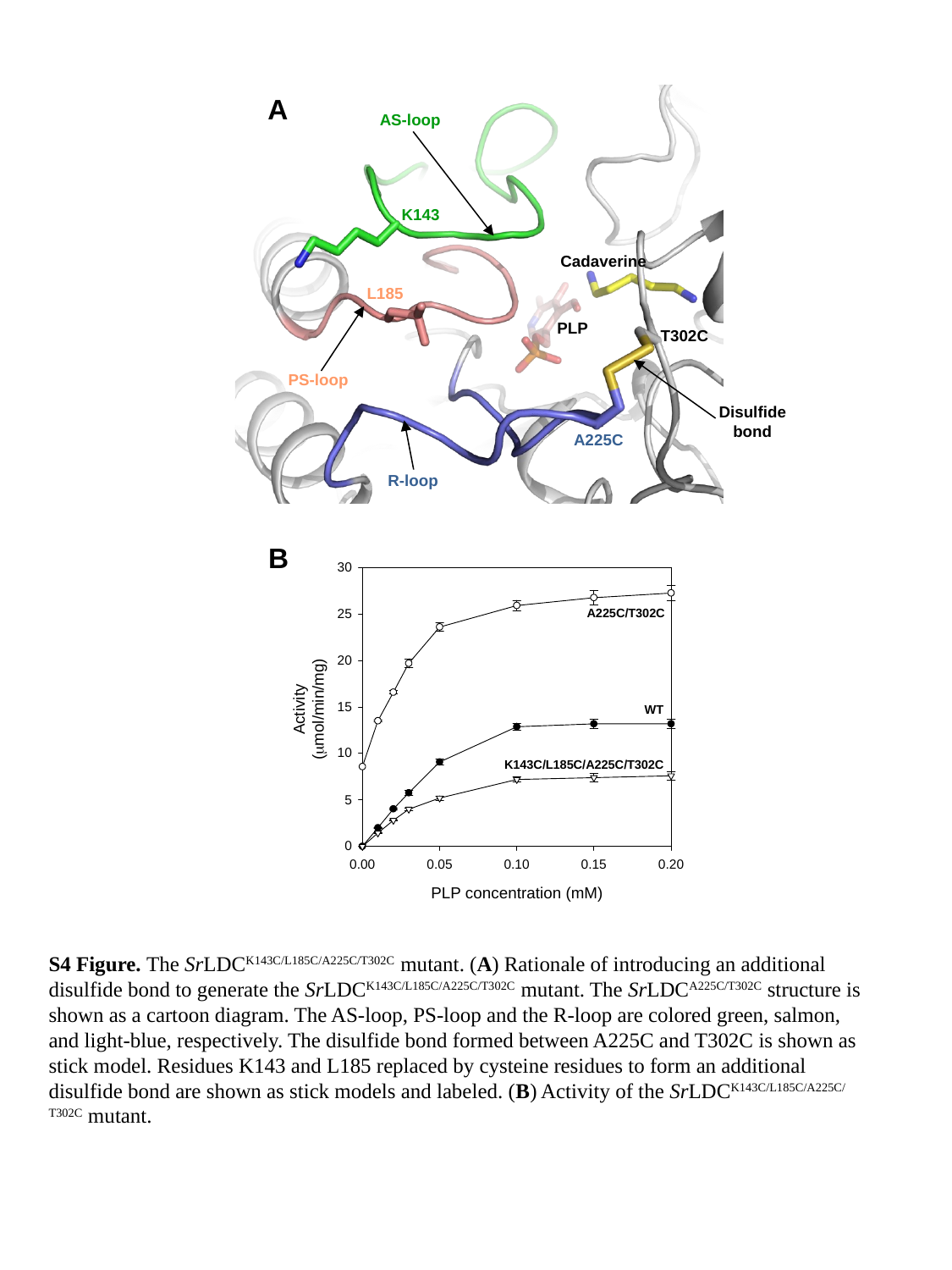

A
AS-loop
K143
Cadaverine
L185
PLP
T302C
PS-loop
Disulfide
bond
A225C
R-loop
B
A225C/T302C
Activity
(mmol/min/mg)
WT
K143C/L185C/A225C/T302C
PLP concentration (mM)
S4 Figure. The SrLDCK143C/L185C/A225C/T302C mutant. (A) Rationale of introducing an additional disulfide bond to generate the SrLDCK143C/L185C/A225C/T302C mutant. The SrLDCA225C/T302C structure is shown as a cartoon diagram. The AS-loop, PS-loop and the R-loop are colored green, salmon, and light-blue, respectively. The disulfide bond formed between A225C and T302C is shown as stick model. Residues K143 and L185 replaced by cysteine residues to form an additional disulfide bond are shown as stick models and labeled. (B) Activity of the SrLDCK143C/L185C/A225C/T302C mutant.
