# Supplementary material for: Lysine Decarboxylase with an Enhanced Affinity for Pyridoxal 5-Phosphate by Disulfide Bond-Mediated Spatial Reconstitution
Source: PLoS One. 2017 Jan 17;12(1):e0170163. doi: 10.1371/journal.pone.0170163 (PMC5240995; doi:10.1371/journal.pone.0170163)
Supplement: S1 Table — (PPTX) [file pone.0170163.s005.pptx]

## Slide 1
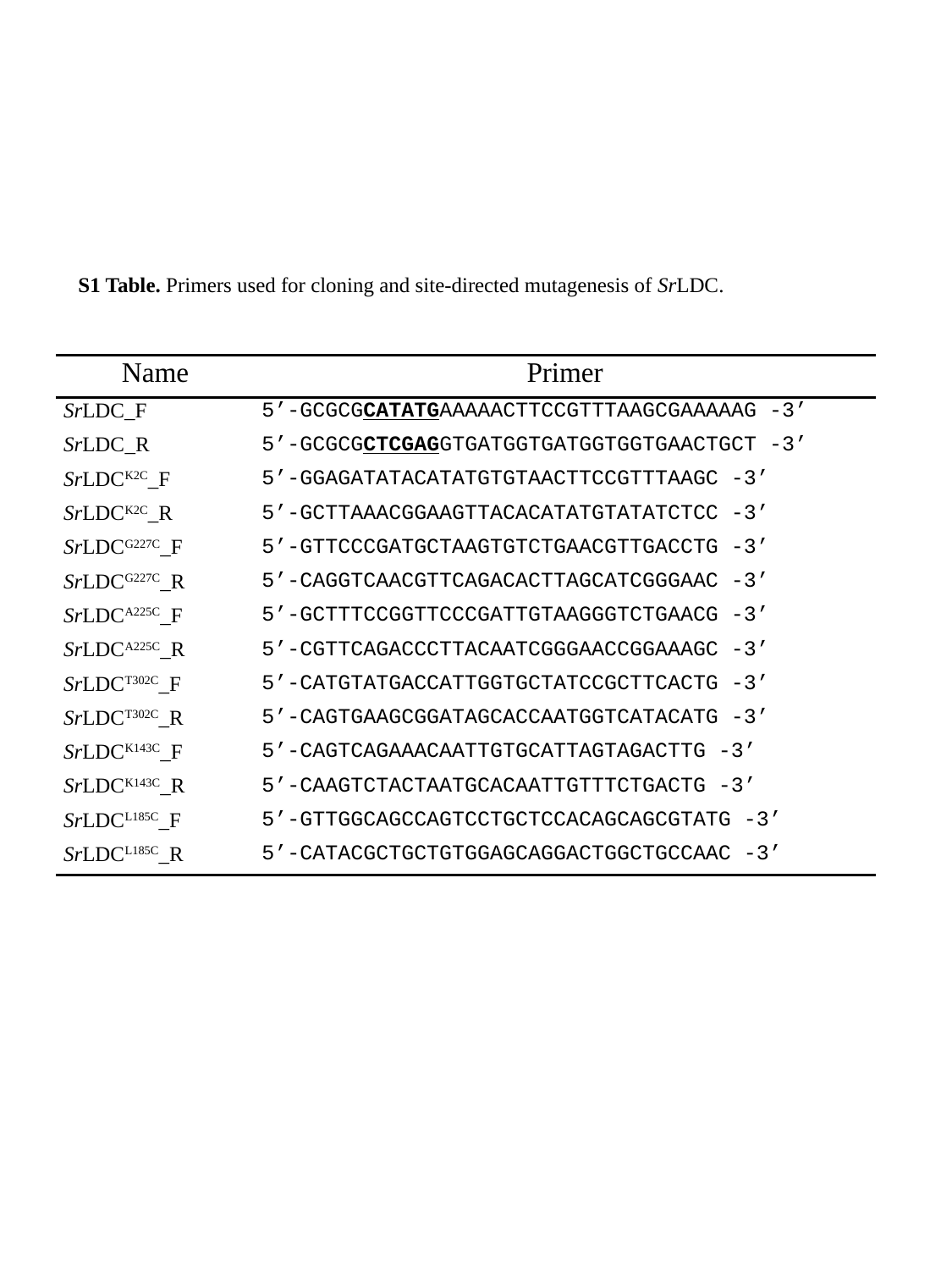

S1 Table. Primers used for cloning and site-directed mutagenesis of SrLDC.
| Name | Primer |
| --- | --- |
| SrLDC\_F | 5’-GCGCGCATATGAAAAACTTCCGTTTAAGCGAAAAAG -3’ |
| SrLDC\_R | 5’-GCGCGCTCGAGGTGATGGTGATGGTGGTGAACTGCT -3’ |
| SrLDCK2C\_F | 5’-GGAGATATACATATGTGTAACTTCCGTTTAAGC -3’ |
| SrLDCK2C\_R | 5’-GCTTAAACGGAAGTTACACATATGTATATCTCC -3’ |
| SrLDCG227C\_F | 5’-GTTCCCGATGCTAAGTGTCTGAACGTTGACCTG -3’ |
| SrLDCG227C\_R | 5’-CAGGTCAACGTTCAGACACTTAGCATCGGGAAC -3’ |
| SrLDCA225C\_F | 5’-GCTTTCCGGTTCCCGATTGTAAGGGTCTGAACG -3’ |
| SrLDCA225C\_R | 5’-CGTTCAGACCCTTACAATCGGGAACCGGAAAGC -3’ |
| SrLDCT302C\_F | 5’-CATGTATGACCATTGGTGCTATCCGCTTCACTG -3’ |
| SrLDCT302C\_R | 5’-CAGTGAAGCGGATAGCACCAATGGTCATACATG -3’ |
| SrLDCK143C\_F | 5’-CAGTCAGAAACAATTGTGCATTAGTAGACTTG -3’ |
| SrLDCK143C\_R | 5’-CAAGTCTACTAATGCACAATTGTTTCTGACTG -3’ |
| SrLDCL185C\_F | 5’-GTTGGCAGCCAGTCCTGCTCCACAGCAGCGTATG -3’ |
| SrLDCL185C\_R | 5’-CATACGCTGCTGTGGAGCAGGACTGGCTGCCAAC -3’ |
